# Actionable Pharmacogenetic Variation in the Slovenian Genomic Database

**DOI:** 10.3389/fphar.2019.00240

**Published:** 2019-03-14

**Authors:** Keli Hočevar, Aleš Maver, Borut Peterlin

**Affiliations:** Clinical Institute of Medical Genetics, University Medical Centre Ljubljana, Ljubljana, Slovenia

**Keywords:** next-generation sequencing, pharmacogenomics, personalized medicine, Slovenian population, PharmGKB

## Abstract

**Background:** Genetic variability in some of the genes that affect absorption, distribution, metabolism, and elimination (“pharmacogenes”) can significantly influence an individual’s response to the drug and consequently the effectiveness of treatment and possible adverse drug events. The rapid development of sequencing methods in recent years and consequently the increased integration of next-generation sequencing technologies into the clinical settings has enabled extensive genotyping of pharmacogenes for personalized treatment. The aim of the present study was to investigate the frequency and variety of potentially actionable pharmacogenetic findings in the Slovenian population.

**Methods:** De-identified data from diagnostic exome sequencing in 1904 cases submitted to our institution were analyzed for variants within 293 genes associated with drug response. Filtered variants were classified according to population frequency, variant type, the functional impact of the variant, pathogenicity predictions and characterization in the Pharmacogenomics Knowledgebase (PharmGKB) and ClinVar.

**Results:** We observed a total of 24 known actionable pharmacogenetic variants (PharmGKB 1A or 1B level of evidence), comprising approximately 26 drugs, of which, 12 were rare, with the population frequency below 1%. Furthermore, we identified an additional 61 variants with PharmGKB 2A or 2B clinical annotations. We detected 308 novel/rare potentially actionable variants: 177 protein-truncating variants and 131 missense variants predicted to be pathogenic based on several pathogenicity predictions.

**Conclusion:** In the present study, we estimated the burden of pharmacogenetic variants in nationally based exome sequencing data and investigated the potential clinical usefulness of detected findings for personalized treatment. We provide the first comprehensive overview of known pharmacogenetic variants in the Slovenian population, as well as reveal a great proportion of novel/rare variants with a potential to influence drug response.

## Introduction

Genetic variation in genes associated with drug pharmacokinetics (i.e., absorption, distribution, metabolism, elimination) or pharmacodynamics (e.g., alerting a drug’s target or perturbing biological pathways) can significantly contribute to individual responsiveness to drugs, and thus on the therapeutic efficacy and toxicity (Dunnenberger et al., 2015; Relling and Evans, 2015). As an integral part of personalized medicine, pharmacogenomics has great potential to enhance clinical benefit, decrease adverse drug reactions and cost of treatment by optimizing drug selection and dosing for an individual. The rapidly dropping cost of next-generation sequencing within recent years, and consequently, the increased integration of these technologies into the clinical settings offered an unprecedented opportunity for extensive genotyping of pharmacogenes. Exome sequencing is a powerful tool for gaining insight into both common and rare coding variation. However, presently, there are no comprehensive studies regarding the application of exome sequencing data for reporting of pharmacogenetic variants. Therefore, there are two challenges that arise while analyzing exome-sequencing data. First, how and which variants with established pharmacogenomic effects should be reported, and second, how to evaluate novel putatively functional variants or variants in genes with less established pharmacogenetic function.

The lack of a comprehensive overview of the distribution of rare variants in pharmacogenes among different ethnic groups and the lack of knowledge about their functional consequences and clinical actionability additionally limit the fully integrated use of pharmacogenomics in a routine clinical practice. Therefore, pharmacogenomics usually remains focused on a limited number of common variants in a small number of genes, the detection of which is primarily based on targeted gene panels or genotyping arrays, such as AmpliChip CYP450 test (Roche) or Affymetrix DMET Plus Assay (Potamias et al., 2014). However, these approaches do not consider the complete heterogeneity of the variation within pharmacogenes and do not address the issue of rare and private variants with potentially large effects. Moreover, previous studies have shown that the vast majority of protein-coding variation is rare, previously unknown, population-specific and enriched for deleterious alleles (Nelson et al., 2012; Tennessen et al., 2012; Gordon et al., 2014; Fujikura et al., 2015). Thus, it is likely that rare variation importantly contributes to some currently unexplained differences in pharmacological responsiveness and metabolism. Consistent with this notion, recent research highlighted that rare variants account for 30–40% of the functional variability in the pharmacogenes (Kozyra et al., 2016). In the study by Ramsey et al. (2012), authors showed that rare variants account for 17.8% of the variability attributed to *SLCO1B1*, a gene associated with methotrexate clearance and disposition of many other medications including statins and irinotecan.

With an objective to enable the clinical use of pharmacogenomics, projects like eMERGE are systematically documenting and evaluating both common and rare variants in pharmacogenes, thus creating clinically useful electronic networks of pharmacogenetic variation (Rasmussen-Torvik et al., 2014). Additionally, the Clinical Pharmacogenetics Implementation Consortium (CPIC ^1^) (Caudle et al., 2014), a shared project between the Pharmacogenomics Knowledgebase (PharmGKB ^2^) (Whirl-Carrillo et al., 2012) and the Pharmacogenomics Research Network (PGRN) (Shuldiner et al., 2013), started to develop peer-reviewed, evidence-based guidelines for specific gene/drug combinations. By September 2018 CPIC published 65 dosing guidelines covering 15 genes and 38 drugs ^3^. The efforts to facilitate implementation have also been undertaken by other nationwide networks such as the Royal Dutch Association for the Advancement of Pharmacy and Canadian Pharmacogenomics Network for Drug Safety (Ross et al., 2010). Currently, 23 different genes have described actionable variants (corresponding to PharmGKB level 1A or 1B of evidence) for germline pharmacogenomics (last accessed on September 9th, 2018).

However, there are presently no consensus recommendations on which pharmacogenetic findings should be actively sought and reported back to patients when analyzing exome or genome sequencing data. Nevertheless, the potential usefulness of pharmacogenomic findings in the exome sequencing data has recently been implicated. In the study of Lee et al. (2016), 21 potentially useful PharmGKB actionable variants (1A and 1B) were identified in 645 individuals who have undergone clinical exome sequencing. In a related study by Cousin et al. (2017), secondary pharmacogenetic findings from clinical whole exome sequencing (WES) testing were reported in a cohort of 94 primarily pediatric patients referred for a suspected genetic disorder. The study results showed that 91% of patients had at least one pharmacogenetic variant allele in *CYP2C19*, *CYP2C9*, and *VKORC1* genes and that 20% of them had potential immediate implications on current medication use. A study on 60,706 human exomes from ExAC population dataset further estimated the prevalence of common as well as rare functional variants in 806 drug-related genes and its implications for 1236 FDA approved drugs. The extended exome data analysis revealed that four in five patients are likely to carry a variant with possibly functional effects (Schärfe et al., 2017).

As population data are specific and cannot be generalized, even within closely related European populations (Mizzi et al., 2017), we used a genomic database of 1904 Slovenian individuals to comprehensively assess the population burden of pharmacogenetic variants. We conducted a nationally based survey of genetic variation within 293 genes, known to influence drug response. Our additional motivation was to assess the applicability of the pharmacogenetic reporting as a part of the routine analysis of exomes and to gain insight into the opportunities and challenges that arise. Accordingly, we analyzed (1) which pharmacogenomic variants could be covered with exome sequencing data, (2) the frequency of known actionable variants in the Slovenian population, (3) and the frequency of rare variants with putative functional impacts and the possibilities for their interpretation.

## Materials and Methods

### Participants

Exome datasets from 1904 patients who were referred to the Clinical Institute of Medical Genetics, University Medical Centre, Ljubljana, Slovenia from July 2014 to October 2017, and have undergone clinical (Illumina TruSight One panel, targeting 4813 genes associated with Mendelian disorders) or whole exome sequencing (Agilent SureSelect All Exon V5 or Illumina Nextera coding exome capture), were recruited for this analysis. All patients gave informed consent for participation in accordance with the Declaration of Helsinki. The study was approved by the Slovenian National Medical Ethics Committee (0120-561/2016). Data were de-identified and phenotype data of the patients were not available.

### Panel Design

The panel of genes analyzed in the present study was selected based to include genes that were captured with all of the used protocols (Illumina TruSight One protocol, Nextera Coding Exome, and the Agilent SureSelect All Exon v5 protocol). We have established the pharmacogenetic list of 293 genes associated with pharmacological impacts, which is based on 33 genes from VeraCode^®^ ADME Core Panel Assay (Illumina) and supplemented with 260 additional genes from PharmaADME (198 genes), PharmGKB (37 genes), and eMERGE-PGx (Sphinx) (25 genes) websites (Supplementary Table S1). Combining clinically relevant genes from these sources ensures that our gene set covers the majority of the key genes currently reviewed in pharmacogenomics research that are also captured with both – the clinical and whole exome sequencing.

### Exome Sequencing

Of the 1904 samples sequenced, the majority (1,582 samples, 83.1%) of the samples were enriched using the Illumina TruSight One protocol, followed by Nextera Coding Exome (188 samples, 9.9%) and the remaining were analyzed using the Agilent SureSelect All Exon v5 (134 samples, 7.0%) protocol. Raw sequence files were processed using a custom exome analysis pipeline, based upon GATK best practices backbone. Reads were aligned to UCSC hg19 human reference genome assembly using Burrows-Wheeler (BWA) algorithm and duplicate sequences were removed using Picard MarkDuplicates, followed by base quality score recalibration, variant calling, variant quality score recalibration, and variant filtering using elements of the GATK toolset (Depristo et al., 2011). In all cases, we attained a minimum median exome coverage of 60x, with over 95% of targets covered with at least 10× sequencing depth. Although the cytochrome genes are characterized by a high degree of homology, we were able to uniquely map over 90% of the reads in these regions, while the non-uniquely mapped reads were attributed mapping quality of 0 by the BWA. GATK variant caller did not emit sequence variants in these regions, thereby reducing the rate of low-quality variants in regions of high homology.

### Variant Analysis

Variants were stored and annotated in our in-house variant collection and annotation system, which is based on vTools software. Variant effect predictions were made using snpEff (Cingolani et al., 2012) and ANNOVAR tools (Wang et al., 2010) and were based on RefSeq gene models (O’Leary et al., 2016), whereas annotations from dbSNP v141 were used for single nucleotide polymorphism (SNP) annotation. Genome Aggregation Database (gnomAD) (Lek et al., 2016) was employed as a source of variant frequencies in worldwide populations. The consensus calls of dbNSFP v2 (Liu et al., 2013) precomputed pathogenicity predictions were used to predict functional effect for missense variants, including SIFT (Sim et al., 2012), Polyphen-2 (Adzhubei et al., 2010), MutationTaster (Schwarz et al., 2014), CADD (Combined Annotation–Dependent Depletion Score) (Kircher et al., 2014), and MetaSVM (Dong et al., 2015). GERP++ rejected substation (RS) scores were used as the source of information for evolutionary sequence conservation applicable to all types of variants (Davydov et al., 2010). Our pipeline included ClinVar as a source of known disease or drug response association of identified variants. Variants that reached coverage less than 20 and quality less than 300 were excluded from the subsequent analysis.

### Variant Filtration and Characterization

Firstly, we applied a 293-gene panel for the filtration of exome data. Next, variants were characterized according to PharmGKB levels of evidence for variant-drug associations ^4^ (Whirl-Carrillo et al., 2012) (accessed on 9th September 2018). Level 1A category includes variant-drug pairs with a CPIC pharmacogenetic guideline or variants implemented at a PGRN site or another major health system. Level 1B annotations comprise variant-drug combinations in which the preponderance of evidence shows an association that has been replicated in more than one cohort, with significant *p*-values and preferably with a strong effect size. Clinical annotation of Level 2A refers to variants within known pharmacogenes that are more likely to have a functional significance. Level 2B annotation refers to variant-drug pairs with moderate evidence of an association that has been replicated, but the results might not be statistically significant or the effect size may be small. Initially, we searched for actionable variants, defined as variants with PharmGKB 1A and 1B levels of evidence. The search was based on dbSNP accession numbers. Star allele assignments (ec. CYP2C9^∗^3) were searched for corresponding rs numbers where possible, using star-allele nomenclature from PharmVar Database ^5^ and TPMT Nomenclature Committee websites ^6^. Additionally, we extracted variant-drug pairs with PharmGKB annotations of 2A or 2B. We also inspected how many variants with ClinVar accession “drug response” were detected in our dataset. The dosing algorithms were obtained from CPIC guidelines and variant-drug-phenotype associations from PharmGKB website ^7^.

Next, we filtered variants on the basis of their minor allele frequencies (MAFs), with an exclusion of variants with MAF > 0.01 in the gnomAD database. We also excluded variants that were detected in more than 19 (1%) individuals as heterozygous and variants detected in more than 15 individuals as homozygous in the Slovenian genomic database. We rated the variants according to variant functional impact, variant type, and theoretical pathogenicity predictions (PolyPhen-2, SIFT, Mutation Tester, MetaSVM, CADD). Additionally, median Phred normalized CADD annotation scores for each gene were calculated (Kircher et al., 2014). Subsequently, we examined the distribution of rare exonic and splicing variation across major pharmacogenetic gene groups, including cytochrome P450 (CYP) superfamily, ATP-binding cassette (ABC) superfamily, solute carrier (SLC) superfamily, and UDP-glucuronosyltransferases (UGTs).

## Results

### Overview

**FIGURE 1:**
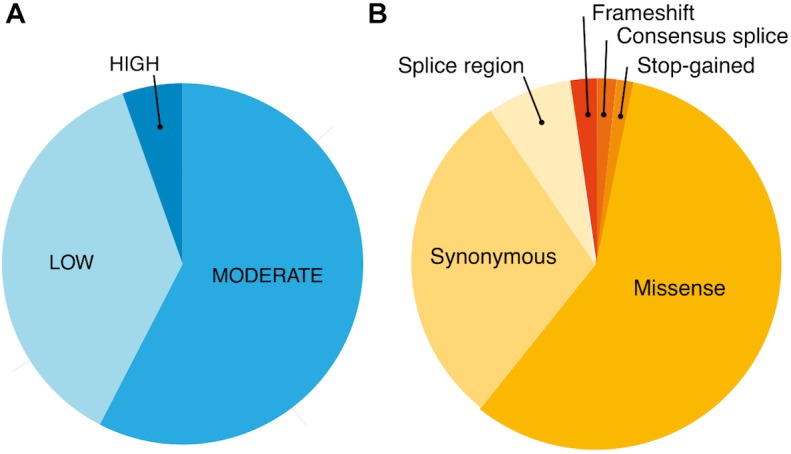
Counts by variant annotation impact (SNPEfff) **(A)** and variant annotation type (SNPEfff) **(B)** for rare exonic and splicing variation in 293 pharmacogenes (MAF < 0.01).

**FIGURE 2:**
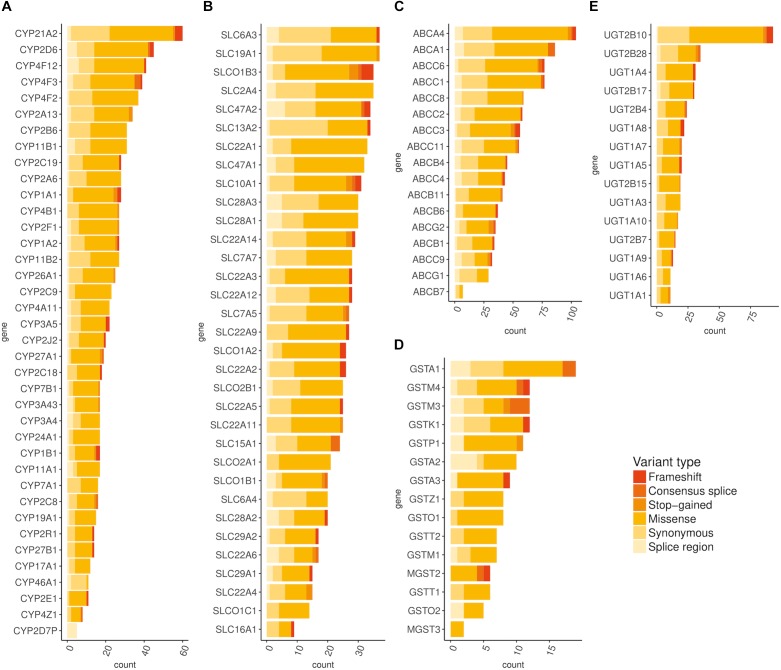
Distribution of rare (MAF < 0.01) exonic and splicing variation across major pharmacogenetic gene groups. **(A)** Distribution of rare exonic and splicing variation across CYP genes, **(B)** Distribution of rare exonic and splicing variation across SLC genes, **(C)** Distribution of rare exonic and splicing variation across ABC genes, **(D)** Distribution of rare exonic and splicing variation across ABC genes, **(E)** Distribution of rare exonic and splicing variation across UGT genes.

Using exome-sequencing data from 1904 individuals we detected a total of 72,293 high-quality variants in 293 pharmacogenes. Our data revealed that most of the variants in pharmacogenes were rare (*n* = 65,059, MAF_gnomAD_ < 0.01), comprising about 90.0% of all variants. Of these rare variants 4360 were annotated as missense, 2239 as synonymous, 174 as frameshifts, and 127 as stop gained. Among the rare non-coding variants, 48,822 were classified as intronic, 1142 as upstream variants, 700 as downstream variants, 1914 as 3′UTR variants, and 735 as 5′UTR variants. Of the rare variants, 9229 (14.2%) were previously reported in the gnomAD database. The number of variants by type is summarized in Table 1. The counts by variant annotation impact and variant annotation type for rare exonic and splicing variation are presented in Figure 1. The distribution of rare variation across major pharmacogenetic gene groups is presented in Figure 2.

**Table 1:** SNPEff Variant types.

	In Slovenian genomic dataset	In gnomAD	In dbSNP 141	Novel (not in gnomAD, dbSNP or ClinVar)
All variants	72293	10646	3083	59428
Rare variants (MAF_gnomAD_ < 0.01), number of heterozygotes < 19, number of homozygotes < 15	61060	9229	2269	51393
Intronic rare	48822	2703	20	46087
Upstream rare	1142	70	0	1070
Downstream rare	700	58	0	641
3′UTR rare	1914	286	2	1354
5′UTR rare	735	155	3	528
Exonic + splicing rare	7571	5831	2225	1669
Missense rare	4360	3394	2079	920
Frameshift rare	174	81	0	86
Synonymous rare	2239	1786	71	448
Splice Acceptor rare	48	24	9	23
Splice Donor rare	60	38	13	21
Stop Gain rare	127	80	48	40
Splice region rare	563	428	6	135
Rare CADD > 20	2101	1615	990	437
Missense rare 4xD + CADD > 20	565	420	260	131
HIGH impact rare (truncating variants)	429	234	76	177
ClinVar at least one 6 (all)	89	70		
ClinVar at least one 6 (rare)	16	9		
PharmGKB Level 1A/1B (all)	24	23		
PharmGKB Level 1A/ 1B (rare)	12	12		
PharmGKB Level 2A/2B (all)	68	50		
PharmGKB Level 2A/ 2B (rare)	12	4		


*gnomAD, Genome Aggregation Database; MAF, minor allele frequency; PharmGKB, Pharmacogenomics Knowledgebase.*

### Known Actionable Pharmacogenetic Variants

Firstly, we focused on variants featured in the PharmGKB or/and in ClinVar database. We looked for PharmGKB annotated variant-drug combinations with four highest levels of evidence 1A, 1B, 2A, and 2B. Within the exome sequencing data we identified 24 PharmGKB unique variants with the highest levels of evidence, 1A or 1B, associated with response to about 26 drugs. Twelve of them were rare, with the MAF not exceeding 1% in gnomAD and the Slovenian genomic database. Rare actionable variants (PharmGKB 1A or 1B) located in the exonic or splicing regions were observed in the following genes: *CYP2D6* (frameshift variant), *CYP2C19* (one start lost and another missense variant), *CFTR* (disruptive inframe deletion and five missense variants), *DYPD* (one missense variant and another splice donor variant), and *TPMT* (one missense variant).

We estimated minor allele frequencies for the Slovenian population (MAF_Slo_) for each potentially actionable finding and the results are presented in Table 2 and Supplementary Tables S2–S4. The most prevalent actionable variant (PharmGKB 1A or 1B level) in our database was a missense variant in *CYP4F2* gene (Val433Met, rs2108622) with MAF_Slo_ of 27.4%, associated with warfarin dosage (PharmGKB level 1A of evidence), followed by missense variants in *CYP2B6* (Gln172His, rs3745274, MAF_Slo_ = 22.4%, PharmGKB 1B), *CYP2D6* (Pro34Ser, rs1065852, MAF_Slo_ = 19.5%, PharmGKB 1A), and *SLCO1B1* (Val174Ala, rs4149056, MAF_Slo_ = 19.2%, PharmGKB 1A) genes. Further most prevalent variants in PharmGKB 1A or 1B category were splice acceptor variant (c.506-1G > A, rs3892097) in *CYP2D6* gene (MAF_Slo_ = 16.7%) and synonymous variant (Pro227Pro, rs4244285) in *CYP2D6* gene (MAF_Slo_ = 12.6%).

**Table 2:** Variant-drug pairs with 1A or 1B clinical annotation according to Pharmacogenomics Knowledgebase (PharmGKB).

Gene	Variant type	Transcript level variant	Functional effect	dbSNP	MAF_gnomAD_	Het_Slo_	MAF_Slo_	Hom_Slo_	PharmGKB	Drugs
*CYP2C9* (^∗^3)	Missense	c.1075A > C	Ile359Leu	1057910	0.0636	247	0.0691	8	1A	Warfarin, phenytoin
*CYP2C9* (^∗^2)	Missense	c.430C > T	Arg144Cys	1799853	0.0926	390	0.121	35	1A	Warfarin, phenytoin
*CYP2D6* (^∗^4)	Splice acceptor	c.506-1G > A	–	3892097	0.138	487	0.167	74	1A	Amitriptyline, clomipramine, desipramine, doxepin, imipramine, nortriptyline, trimipramine
*CYP2D6* (^∗^6) (non-functional)	Frameshift	c.454delT	Trp152fs	5030655	0.00791	42	0.0110	0	1A	Paroxetine, fluvoxamine, amitriptyline, nortriptyline, codeine
*CYP2D6* (all the variants in which it appears have reduced or no *CYP2D6* activity)	Missense	c.100C > T	Pro34Ser	1065852	0.207	511	0.195	115	1A	Paroxetine, nortriptyline, codeine, amitriptyline
								1B	Tramadol
*SLCO1B1* (^∗^5)	Missense	c.521T > C	Val174Ala	4149056	0.133	585	0.192	73	1A	Simvastatin
*CYP2D6* (^∗^3)	Frameshift	c.775delA	Arg259fs	35742686	0.0124	67	0.0186	2	1A	Amitriptyline, nortriptyline, trimipramine, clomipramine, tamoxifen, codeine, paroxetine, doxepin, fluvoxamine
								1B	Tramadol
*VKORC1*	3′-UTR	c.^∗^134G > A	–	7294	.	64	0.0273	20	1B	Warfarin
*CYP4F2*	Missense	c.1297G > A	Val433Met	2108622	0.274	759	0.274	142	1A	Warfarin
*CYP2B6*	Missense	c.516G > T	Gln172His	3745274	0.272	651	0.224	101	1B	Efavirenz
*CYP2C19* (^∗^2)	Synonymous	c.681G > A	Pro227Pro	4244285	0.176	411	0.126	35	1A	Amitriptyline, clopidogrel
*CYP2C19* (^∗^4)	Start lost	c.1A > G	Met1?	28399504	0.00231	11	0.00289	0	1A	Clopidogrel
*CYP2C19* (^∗^8) (non-functional)	Missense	c.358T > C	Trp120Arg	41291556	0.00152	11	0.00289	0	1A	Clopidogrel
*CFTR*	Disruptive inframe deletion	c.1521_1523delCTT	Phe508del	113993960	0.00696	39	0.0102	0	1A	Ivacaftor
*CFTR*	Missense	c.220C > T	Arg74Trp	115545701	0.00142	1	0.000263	0	1A	Ivacaftor
*CFTR*	Missense	c.328G > C	Asp110His	113993958	0.0000203	1	0.000263	0	1A	Ivacaftor
*CFTR*	Missense	c.3154T > G	Phe1052Val	150212784	0.000632	4	0.00105	0	1A	Ivacaftor
*CFTR*	Missense	c.3209G > A	Arg1070Gln	78769542	0.000623	1	0.000263	0	1A	Ivacaftor
*CFTR*	Missense	c.3454G > C	Asp1152His	75541969	0.000407	2	0.000526	0	1A	Ivacaftor
*DYPD*	Missense	c.2846A > T	Asp949Val	67376798	0.00284	9	0.00236	0	1A	Capecitabine, fluorouracil, pyrimidine analogs, tegafur
*DPYD* (^∗^2A)	Splice donor	c.1905+1G > A	–	3918290	0.00574	10	0.00263	0	1A	Capecitabine, fluorouracil, pyrimidine analogs, tegafur
*TPMT* (^∗^2)	Missense	c.238G > C	Ala80Pro	1800462	0.00172	6	0.00158	0	1A	Azathioprine, mercaptopurine, purine analogs, thioguanine
*TPMT* (^∗^3B/potentially encoding ^∗^3A allele)	Missense	c.460G > A	Ala154Thr	1800460	0.0280	93	0.0249	1	1A	Azathioprine, mercaptopurine, purine analogs, thioguanine
*TPMT* (potentially encoding ^∗^3C allele /^∗^3A)	Missense	c.719A > G	Tyr240Cys	1142345	0.0366	98	0.0268	2	1A	Azathioprine, mercaptopurine, purine analogs, thioguanine


*Het_Slo_, number of heterozygotes in the Slovenian genomic database; Hom_*Slo*_, number of homozygotes in Slovenian genomic database; MAF, minor allele frequency; 1A, variant-drug pairs with a CPIC pharmacogenetic guideline or variants implemented at a PGRN site or another major health system; 1B, variant-drug pairs in which the preponderance of evidence shows an association that has been replicated in more than one cohort, with significant p-values and preferably with a strong effect size.*

Additionally, we identified 68 variants with PharmGKB 2A or 2B levels of evidence, with seven of them also in the 1st categories (PharmGKB 1A and 1B), but presented a different type of an association or different drug-variant pair and were for that reason listed twice. Altogether, the most common pharmacogenetic variant detected in the Slovenian genomic database was a missense variant in the *F5* gene (Gln534Arg, rs6025, PharmGKB 2A) with MAF_Slo_ of 88.4% (MAF_gnomAD_ = 98.0%). This was followed by a synonymous variant in *ABCC4* gene (Lys1116Lys, rs1751034, PharmGKB 2B), associated with a response to tenofovir and MAF_Slo_ of 77.0% (MAF_gnomAD_ = 81.0%). A missense variant in the *TP53* gene (Pro72Arg, rs1042522, PharmGKB 2B) with MAF in the Slovenian population of 71.8% (MAF_gnomAD_ = 66.9%) was the third most frequently detected pharmacogenetic variant in Slovenian individuals. The variant is associated with the efficacy and toxicity/ADR of antineoplastic agents, such as cisplatin, cyclophosphamide, fluorouracil, and paclitaxel. A start lost variant in *VDR* gene (Met1? rs2228570) was the second most prevalent variant in the PharmGKB 2A category, with MAF_Slo_ of 52.8% (MAF_gnomAD_ = 62.9%). The variant is associated with the efficacy in response to peginterferon alfa-2b in patients suffering from chronic hepatitis C. A missense variant in *COMT* gene (Val158Met, rs4680, PharmGKB 2A) reached the MAF_Slo_ of 48.6%, which is in line with gnomAD MAF frequency of 46.3%. Variant-drug pairs along with corresponding frequencies are summarized in Table 2 and Supplementary Tables S2–S4.

We detected 89 ClinVar variants with at least one accession number 6 (“drug response”), 16 of them with MAF of less than 1% (ClinVar version 02.10.2017).

When we compared MAFs for each risk variant in the Slovenian genomic database to MAFs in the gnomAD database, we generally got consistent results for detected exonic and splicing variation. However, some inconsistencies in the MAFs among databases were apparent. For example, the variant in *ANKK1* gene (Glu713Lys, rs1800497) associated with the toxicity and ADR of antipsychotics, had a MAF of 26.4% in the gnomAD database (MAF_European(Non-Finnish)_ = 19.2%), but had a MAF of only 17.3% in the Slovenian genomic database. A synonymous variant in the *CYP2C19* gene (Pro227Pro, rs4244285) influencing the efficacy of several drugs including amitriptyline, clopidogrel, citalopram, and clomipramine, had a MAF of in 17.6% in the gnomAD database (MAF_European(Non-Finnish)_ = 14.7%) and 12.6% in the Slovenian database. The variant in *F5* gene (Gln534Arg, rs6025), associated with the adverse event of thrombosis in systemic hormonal contraceptives use, had a MAF of 98.0% in gnomAD (MAF_European(Non-Finnish)_ = 97.0%) and only of 88.4% in Slovenian population. In contrast, missense variant in the *SLCO1B1* gene (Val174Ala, rs4149056) associated with the adverse drug reaction and toxicity of simvastatin, has MAF of 13.3% in gnomAD (MAF_European(Non-Finnish)_ = 15.6%) and 19.2% in the Slovenian database. It is important to note that some of the detected variants were intronic, therefore, their MAF_Slo_ may be unreliable due to the lack of sequence coverage for these regions in exome sequencing data.

### Functional Impacts of Variants

Next, we characterized the rare variants on the basis of predicted functional impacts. We detected 2101 variants that reached CADD score above the cut-off value of 20 and thus ranked into the 1^st^ percentile of the most deleterious variants (Kircher et al., 2014). Several *in silico* prediction algorithms (Mutation Tester, Polyphen-2, SIFT, MetaSVM) predicted in consensus as pathogenic 565 missense variants that also reached CADD score above 20. These included 131 novel variants- not previously reported in gnomAD, dbSNP or ClinVar database (Supplementary Table S5). We further analyzed rare variants with protein-truncating effects, including frameshift variants, stop-gain variants, and variants affecting splicing, which resulted in additional 429 variants predicted to be highly pathogenic based on their functional impact, of which 177 were novel (Supplementary Table S6). Most of the rare putatively functional variants were thus missense, followed by frameshift (*n* = 174) and stop-gain variants (*n* = 127).

Additionally, we examined CADD annotation scores separately for each gene and further compared median CADD scores of rare variants with common variants. We observed the highest median CADD scores for rare variants in the following genes: *CDA*, *SLC10A2*, *TPMT*, and *SULF1*. Among CYP genes, the highest median score was detected in the *CYP1A1* gene, followed by *CYP24A1*, and *CYP2R1*. In the SLC group, *SLC10A2*, *SLC22A2*, and *SLC22A6* genes ranked the highest. Altogether, the highest CADD score of 54 was detected for the known pathogenic variant in *ABCA4* gene (c.6445C > T, Arg2149^∗^, rs61750654), followed by the stop-gain variant in *EPHX2* gene (Arg467^∗^, CADD = 51). Expectedly, we identified significantly more damaging variants among the rare variants than among common variants with the population frequency exceeding 1%.

## Discussion

The implementation of exome sequencing technologies into daily clinical practice makes the prospect of a personalized treatment increasingly available. Here, we showed that by using clinical and whole exome sequencing technologies it is possible to identify not only variants that are causative for patients’ clinical presentation but also a considerable proportion of pharmacogenetics findings with established evidence and potential clinical utility.

Within the study population, we identified a high frequency of well-established examples of common genetic polymorphisms, as well as known rare actionable variants. We detected 24 variants with compelling evidence of pharmacogenetic significance (PharmGKB level 1A or 1B variants) associated with about 26 drugs, where 12 of them were rare, and 61 additional variants with a level 2A or 2B PharmGKB evidence. Our results are consistent with those of the previously published study by Lee et al. (2016), in which authors used combined SNP chip and exome sequence data of 1101 individuals. In their study, 29 variants were detected that ranked in the PharmGKB 1A and 1B categories; 21 of them were detected by exome sequencing technology. Similarly, 22 actionable clinical variants (PharmGKB 1A/1B) were found in 120 pharmacogenes when analyzing 1000 Genomes Phase 3 data of 2540 individuals (Wright et al., 2018).

Furthermore, our results are correlated with the already known fact that rare variants are enriched for deleterious variation. We identified 308 novel variants of potential functional significance, including 131 missense variants (predicted in consensus as pathogenic by functional prediction algorithms: Mutation Tester, Polyphen-2, SIFT, MetaSVM, CADD) and 177 protein-truncating variants. We observed that especially when testing an expanded set of genes, novel putatively functional variants and variants in genes with less established effects represent a considerable challenge in result interpretation and reporting. To date, very few studies have conducted a systematic overview of the distribution and frequency of genetic variation with potentially high impact over a large set of pharmacogenes (Kozyra et al., 2016; Schärfe et al., 2017; Wright et al., 2018). So far, studies examining rare genetic variation have been limited to small sets of genes or on gene groups, such as largely studied cytochrome P450 (CYP) gene family (Gordon et al., 2014; Fujikura et al., 2015). Further evaluation of functional consequences and clinical effects is required to extend our understanding of rare variants. Therefore, a considerable part of the variation in response to treatment still remains unclear and has not yet been integrated into routine clinical practice.

Moreover, we have observed that MAFs of some known variants differ significantly in the Slovenian dataset when compared to gnomAD MAFs. This raises the importance of establishing population specific databases of pharmacogenomics variation. With growing pharmacogenetic databases and increased integration of sequencing technologies into clinical practice, we will also gain additional insight into the rare pharmacogenetic variation. This will make publicly accessible and easily updatable data repositories such as CPIC, PharmGKB, ClinVar, Pharmacogene Variation (PharmVar) Consortium, as well as population-specific databases, essential for the accurate interpretation of pharmacogenomics results along with the subsequent integration of dosing recommendations and guidelines into electronic healthcare record systems.

Also, the identification and reporting of pharmacogenetic findings are in many aspects distinct from reporting of the disease causative variants. The proposed American College of Medical Genetics and Genomics (ACMG) criteria for interpretation of sequence variants are not intended for pharmacogenomic findings (Richards et al., 2015). While the comprehensive phenotyping data could be of particular value when interpreting the putative disease-causative variants, the genotype-phenotype correlation for pharmacogenomic findings is apparent only when the patient is exposed to a specific drug. Furthermore, the results may not be useful at the time of reporting, but only when the particular drug is prescribed to the patient. However, by potential reporting or storing of actionable variants from sequencing data preemptively, they may be available prior the prescription and for a wide range of medications, subsequently influencing decisions about treatment, which could significantly medically benefit the patients (Dunnenberger et al., 2015; Ji et al., 2016).

Compared to approaches targeting only known pharmacogenetics variants, sequencing technologies are beneficial for a number of additional aspects. SNP genotyping assays may be unable to detect low-frequency variants with potential deleterious functional effects. Besides, the response to a majority of the drugs is influenced by several genes, including genes encoding drug metabolizing enzymes, transporters, drug targets, and disease-modifying genes, or by various variants within the same gene, which may not be detected using approaches targeting known pharmacogenetics variants (Relling and Evans, 2015). We have demonstrated that exome sequencing is an effective method for the detection of both rare and common pharmacogenetic variants in a large set of genes under one investigation.

The present study identifies a high number of clinically relevant highly actionable variant-drug associations, with already established dosing guidelines and recommendations applicable for the use in personalized treatment. Here we highlight the potential clinical utility for a selection of variants detected in the Slovenian database.

A decreased function missense variant in the *SLCO1B1* gene (Val174Ala, rs4149056, allele ^∗^5) has a MAF of 19.2% in the Slovenian population. It was identified as heterozygous in 585/1904 (31%) individuals and as homozygous in 73/1904 (4%) individuals, who, therefore, have an intermediate and high myopathy risk, respectively, when receiving simvastatin treatment. Consequently, a lower dose or alternative statin (e.g., pravastatin or rosuvastatin) and routine creatine kinase (CK) surveillance are recommended for these individuals (Wilke et al., 2012; Ramsey et al., 2014).

Variants of *CYP2D6* and *CYP2C19* genes affect the exposure, efficacy, and safety of tricyclic antidepressants (TCAs) (Hicks et al., 2013). A synonymous variant (Pro227Pro, rs4244285) in the *CYP2C19* gene represents no function allele (^∗^2) and thus greatly decreases the conversion of tertiary amines to secondary amines, which may cause a sub-optimal response. The MAF of the variant in the Slovenian population was estimated at 12.6%; 35/1904 (1.8%) individuals carried the variant in the homozygous state, who should avoid the use of tertiary amine and alternative drugs that are not metabolized by *CYP2C19* are recommended. Moreover, we detected c.506-1G > A variant (allele ^∗^4, rs3892097), with anticipated effect on splicing in *CYP2D6* gene, resulting in a greatly reduced metabolism of TCAs to less active compounds. The variant was found in 487/1904 (25.6%) of Slovenian individuals as heterozygous, and in 74/1904 (3.9%) as homozygous. Additionally, the variant also has a major role in the activation of prodrugs such as codeine and tramadol.

Cytochrome P450 CYP2C19 also catalyzes the bioactivation of the antiplatelet prodrug clopidogrel that inhibits the ADP-dependent P2Y_12_ receptor. *CYP2C19* (^∗^2) loss-of-function allele impairs formation of active metabolites (Scott et al., 2013). Both heterozygous 411/1904 (21.6%) and homozygous 35/1904 (1.8%) clopidogrel-treated patients with acute coronary syndromes have significantly reduced platelet inhibition and thus an increased risk for serious adverse cardiovascular events. Alternative antiplatelet medication, such as prasugrel or ticagrelor is strongly recommended in individuals with this variant.

Furthermore, we detected two rare variants with PharmGKB level 1A of evidence, one variant with the effect on splicing (c.1905+1G > A, allele ^∗^2A, rs3918290, MAF_Slo_ = 0.263%) and another missense variant (c.2846A > T, Asp949Val, rs67376798, MAF_Slo_ = 0.236%) in the *DPYD* gene. Heterozygotes for one of the detected variants in the *DPYD* gene have reduced leukocyte dihydropyrimidine dehydrogenase (DPD) activity (at 30–70% that of the normal population) and an increased risk of severe or even lethal drug toxicity when treated with fluoropyrimidine drugs. At least 50% reduction in starting dose is recommended, followed by titration of dose based on toxicity or pharmacokinetic test (Caudle et al., 2013).

Our study also has some limitations. Firstly, the application of exome sequencing for the detection of pharmacogenomics variants is limited (Londin et al., 2014). Because of the lack of coverage of the exome test, we were not able to accurately detect the majority of the intronic variation (e.g., the rs9923231 variant of the *VKORC1* gene). Due to technical limitations, structural variants and repetitive regions were not sufficiently assessed. We also recognize the limitation of the sensitivity of exome sequencing in highly homologous regions of the human genome, including the cytochrome genes (e.g., part of the known actionable variability of the *CYP2D6* gene). We recognize the possibility that we failed to detect a minor part of pharmacogenomic variation due to the limited detection of variants in these regions. Furthermore, in the present study, we did not extend the exome analysis on copy number variation (CNVs). Also, with de-identified data, we could not identify the compound heterozygous states or assess the polygenic effects of variants. Nevertheless, when analyzing each patient’s data separately, it will be possible to include multigenic effects, compound heterozygous states and some of the risk haplotypes with established pharmacogenetic effects in future patient’s records, which will add the considerable value to the exome sequencing results. With such valuable data, we could significantly benefit future patients by increasing the efficacy and decreasing adverse drug responses of pharmacologic treatment.

## Conclusion

In conclusion, our results demonstrate that nationally based exome sequencing data represents a valuable source for identification of pharmacogenetic variants. The direct inclusion of actionable pharmacogenetics findings in patient’s records could significantly improve the outcome in patients who underwent diagnostic exome and genome sequencing. Furthermore, our data provide the first comprehensive overview of the distribution of both rare and common variants within several pharmacogenes and provides first estimates on their prevalence for the Slovenian population. We have shown that testing beyond known polymorphisms is warranted to gain further insight into rare variation and to facilitate more reliable future interpretation and reporting of pharmacogenetic findings. We anticipate that the present dataset will be of great importance for future research and validation of pharmacogenetics variation in the Slovenian population. Based on our results we propose that known pharmacogenetic variants with well-established effects should be a part of every genetic report.

## Author Contributions

BP, KH, and AM contributed to conception and design of the study. KH and AM performed the statistical analysis. KH wrote the first draft of the manuscript. All authors contributed to manuscript revision, read and approved the submitted version.

## Conflict of Interest Statement

The authors declare that the research was conducted in the absence of any commercial or financial relationships that could be construed as a potential conflict of interest.

## Footnotes

1 www.eu-pic.net

2 www.pharmgkb.org

3 www.pharmgkb.org/guidelines

4 www.pharmgkb.org/downloads

5 www.pharmvar.org/genes

6 www.imh.liu.se/tpmtalleles

7 www.pharmgkb.org

## Abbreviations

ABC: ATP-binding cassette
ACMG: American College of Medical Genetics and Genomics
BWA: Burrows-Wheeler algorithm
CADD: Combined Annotation–Dependent Depletion Score
CNV: copy number variation
CPIC: Clinical Pharmacogenetics Implementation Consortium
CYP: cytochrome P450 superfamily
FDA: Food and Drug Administration
MAF: minor allele frequency
MAF_Slo_: minor allele frequencies for the Slovenian population
gnomAD: the Genome Aggregation Database
PGRN: Pharmacogenomics Research Network
PharmGKB: Pharmacogenomics Knowledgebase
SLC: solute carrier
TCAs: tricyclic antidepressants
UGTs: UDP-glucuronosyltransferases
UCSC: University of California Santa Cruz
