# Supplementary material for: Actionable Pharmacogenetic Variation in the Slovenian Genomic Database
Source: Front Pharmacol. 2019 Mar 14;10:240. doi: 10.3389/fphar.2019.00240 (PMC6428035; doi:10.3389/fphar.2019.00240)
Supplement: Supplementary file 4 [file Tables_2-4.docx]

Supplementary Material

Actionable pharmacogenetic variation in the Slovenian genomic database

Keli Hočevar^1^, Aleš Maver^1^, Borut Peterlin^1*^

*** Correspondence:** Borut Peterlin: borut.peterlin@guest.arnes.si

**Supplementary Table S2.** Variant-drug pairs with 1A or 1B clinical annotation according to Pharmacogenomics Knowledgebase (PharmGKB). ADR, adverse drug reaction; PK, pharmacokinetics; Het_Slo_, number of heterozygotes in the Slovenian genomic database; Hom_Slo_, number of homozygotes in Slovenian genomic database; MAF, minor allele frequency; 1A, variant-drug pairs with a CPIC pharmacogenetic guideline or variants implemented at a PGRN site or another major health system; 1B, variant-drug pairs in which the preponderance of evidence shows an

association that has been replicated in more than one cohort, with significant p-values and preferably with a strong effect size.

| **Gene** | **Variant type** | **Transcript level variant** | **Functional effect** | **dbSNP** | **MAF**  **_gnomAD_** | **Het_Slo_**  **MAF_Slo_** | **Hom_Slo_** | **Type** | **PharmGKB** | **Drugs** | **Phenotypes** | **ClinVar**  **(October 2017)** |
| --- | --- | --- | --- | --- | --- | --- | --- | --- | --- | --- | --- | --- |
| *CYP2C9* (*3) | Missense | c.1075A>C | Ile359Leu | 1057910 | 0.0636 | 247  0.0691 | 8 | Dosage, Toxicity/ADR | 1A | warfarin | - | 6\|6\|6\|6\|6\|6\|6\|6\|6\|6\|6\|255 |
|  | | | | | | | | Toxicity/ADR, Metabolism/  PK | 1A | phenytoin | Epilepsy |  |
| *CYP2C9* (*2) | Missense | c.430C>T | Arg144Cys | 1799853 | 0.0926 | 390  0.121 | 35 | Dosage | 1A | warfarin | - | 255\|6\|6\|2 |
|  | | | | | | | | Toxicity/ADR, Metabolism/  PK | 1A | phenytoin | Epilepsy |  |
|  |  |  |  |  |  |  |  | Dosage | 1A | warfarin | Cardiovascular Diseases, Heart Diseases |  |
| *CYP2D6* (*4) | Splice acceptor | c.506-1G>A | - | 3892097 | 0.138 | 487  0.167 | 74 | Dosage, Toxicity/ADR | 1A | amitriptyline, antidepressants, clomipramine, desipramine, doxepin, imipramine, nortriptyline, trimipramine | Depression | 6\|6\|6\|6\|6\|6\|6\|6\|6\|6\|255 |
| *CYP2D6*  (*6)  (non-functioning variant) | Frameshift | c.454delT | Trp152fs | 5030655 | **0.00791** | 42  **0.0110** | 0 | Efficacy, Toxicity/ADR, Metabolism/PK | 1A | paroxetine  fluvoxamine  amitriptyline  nortriptyline  codeine | Depressive Disorder, Major, Mental Disorders, Obsessive-Compulsive Disorder  Pain | 6 |
| *CYP2D6*  (all the variants in which it appears have reduced or no *CYP2D6*  activity) | Missense | c.100C>T | Pro34Ser | 1065852 | 0.207 | 511  0.195 | 115 | Efficacy, Toxicity/ADR, Metabolism/PK | 1A | paroxetine | Depressive Disorder, Major, Mental Disorders, Obsessive-Compulsive Disorder | 6 |
|  | | | | | | | | Efficacy, Toxicity/ADR, Metabolism/PK | 1A | nortriptyline | Depressive Disorder, Major |  |
|  |  |  |  |  |  |  |  | Efficacy, Toxicity/ADR | 1A | codeine | Pain |  |
|  |  |  |  |  |  |  |  | Efficacy, Toxicity/ADR | 1A | amitriptyline | Depressive Disorder, Major, Mental Disorders, Mood Disorders |  |
|  |  |  |  |  |  |  |  | Dosage, Efficacy, Toxicity/ADR | 1B | tramadol | Pain |  |
| *SLCO1B1* (*5) | Missense | c.521T>C | Val174Ala | 4149056 | 0.133 | 585  0.192 | 73 | Toxicity/ADR | 1A | simvastatin | Muscular Diseases, Myopathy, Central Core | 6\|6\|6\|6\|6\|2\|255\|6 |
| *CYP2D6* (*3) | Frameshift | c.775delA | Arg259fs | 35742686 | 0.0124 | 67  0.0186 | 2 | Efficacy,Toxicity/ADR | 1A | amitriptyline | Depressive Disorder, Major, Mental Disorders, Mood Disorders | 6 |
|  | | | | | | | | Efficacy, Toxicity/ADR, Metabolism/PK | 1A | nortriptyline | Depressive Disorder, Major |  |
|  |  |  |  |  |  |  |  | Other | 1A | trimipramine | - |  |
|  |  |  |  |  |  |  |  | Efficacy, Toxicity/ADR | 1A | clomipramine | Depressive Disorder, Major, Obsessive-Compulsive Disorder |  |
|  |  |  |  |  |  |  |  | Efficacy, Metabolism/PK | 1A | tamoxifen | Breast Neoplasms |  |
|  |  |  |  |  |  |  |  | Efficacy, Toxicity/ADR | 1A | codeine | Pain |  |
|  |  |  |  |  |  |  |  | Efficacy, Toxicity/ADR, Metabolism/PK | 1A | paroxetine | Depressive Disorder, Major, Mental Disorders, Obsessive-Compulsive Disorder |  |
|  |  |  |  |  |  |  |  | Efficacy | 1A | doxepin | - |  |
|  |  |  |  |  |  |  |  | Efficacy, Toxicity/ADR, Metabolism/PK | 1A | fluvoxamine | Depressive Disorder, Major, Mental Disorders, Obsessive-Compulsive Disorder |  |
|  |  |  |  |  |  |  |  | Dosage, Efficacy, Toxicity/ADR, Metabolism/PK | 1B | tramadol | Pain |  |
| *VKORC1* | 3'prime-UTR | c.*134G>A | - | 7294 | **.** | 64  0.0273 | 20 | Dosage | 1B | warfarin | - | 6\|6\|6\|2 |
| *CYP4F2* | Missense | c.1297G>A | Val433Met | 2108622 | 0.274 | 759  0.274 | 142 | Dosage | 1A | warfarin | Heart Diseases, Hemorrhage, Intracranial Hemorrhages, Myocardial Infarction, Peripheral Vascular Diseases, Thromboembolism, venous thromboembolism | 6\|6\|6 |
| *CYP2B6* | Missense | c.516G>T | Gln172His | 3745274 | 0.272 | 651  0.224 | 101 | Dosage | 1B | efavirenz | HIV | 6\|6\|6\|6\|6 |
| *CYP2C19* (*2) | Synonymous | c.681G>A | Pro227Pro | 4244285 | 0.176 | 411  0.126 | 35 | Efficacy | 1A | amitriptyline | - | 6\|6\|6\|6\|6\|6\|6\|255 |
|  | | | | | | | | Efficacy,  Toxicity/ADR | 1A | clopidogrel | Acute coronary syndrome,  Cardiovascular Diseases,  Thrombosis |  |
| *CYP2C19* (*4) | Start lost | c.1A>G | Met1? | 28399504 | **0.00231** | 11  **0.00289** | 0 | Efficacy | 1A | clopidogrel | Acute coronary syndrome,  Cardiovascular Diseases | 6\|6\|255 |
| *CYP2C19*  (*8)  (non-functional) | Missense | c.358T>C | Trp120Arg | 41291556 | **0.00152** | 11  **0.00289** | 0 | Efficacy, Toxicity/ADR | 1A | clopidogrel | - | 255 |
| *CFTR* | Disruptive inframe deletion | c.1521_1523delCTT | Phe508del | 113993960 | **0.00696** | 39  **0.0102** | 0 | Efficacy | 1A | ivacaftor | Cystic Fibrosis | 5\|255\|5\|5\|6\|6 |
| *CFTR* | Missense | c.220C>T | Arg74Trp | 115545701 | **0.00142** | 1  **0.000263** | 0 | Efficacy | 1A | ivacaftor | Cystic Fibrosis | 255\|4\|4\|4\|0 |
| *CFTR* | Missense | c.328G>C | Asp110His | 113993958 | **0.0000203** | 1  **0.000263** | 0 | Efficacy | 1A | ivacaftor | Cystic Fibrosis | 5\|1 |
| *CFTR* | Missense | c.3154T>G | Phe1052Val | 150212784 | **0.000632** | 4  **0.00105** | 0 | Efficacy | 1A | ivacaftor | Cystic Fibrosis | 255\|5 |
| *CFTR* | Missense | c.3209G>A | Arg1070Gln | 78769542 | **0.000623** | 1  **0.000263** | 0 | Efficacy | 1A | ivacaftor | Cystic Fibrosis | 255\|5\|5 |
| *CFTR* | Missense | c.3454G>C | Asp1152His | 75541969 | **0.000407** | 2  **0.000526** | 0 | Efficacy | 1A | ivacaftor | Cystic Fibrosis | 5\|5\|5\|5\|5 |
| *DYPD* | Missense | c.2846A>T | Asp949Val | 67376798 | **0.00284** | 9  **0.00236** | 0 | Toxicity/ADR, Metabolism/  PK | 1A | capecitabine,  fluorouracil,  Pyrimidine analogues,  tegafur | Neoplasms | 1\|6\|6\|6\|4\|6\|6\|6\|6 |
| *DPYD*  (*2A) | Splice donor | c.1905+1G>A | - | 3918290 | **0.00574** | 10  **0.00263** | 0 | Toxicity/ADR, Metabolism/  PK | 1A | capecitabine, fluorouracil, pyrimidine analogues,  tegafur | Neoplasms | 5\|5\|5\|0\|6\|6\|6\|6 |
| *TPMT*2* | Missense | c.238G>C | Ala80Pro | 1800462 | **0.00172** | 6  **0.00158** | 0 | Dosage,  Toxicity/ADR | 1A | azathioprine,  mercaptopurine,  purine analogues,  thioguanine | - | 6 |
| *TPMT* (*3B/potentially encoding *3A allele) | Missense | c.460G>A | Ala154Thr | 1800460 | 0.0280 | 93  0.0249 | 1 | Dosage,  Toxicity/ADR | 1A | azathioprine,  mercaptopurine,  purine analogues,  thioguanine | - | 6\|6\|255 |
| *TPMT* (potentially encoding *3C allele /*3A) | Missense | c.719A>G | Tyr240Cys | 1142345 | 0.0366 | 98  0.0268 | 2 | Dosage,  Toxicity/ADR | 1A | azathioprine,  mercaptopurine,  purine analogues,  thioguanine | - | 6\|6\|255 |

**Supplementary Table S3.** Variant-drug pairs with 2A clinical annotation according to Pharmacogenomics Knowledgebase (PharmGKB). ADR, adverse drug reaction; PK, pharmacokinetics; Het_Slo_, number of heterozygotes in the Slovenian genomic database; Hom_Slo_, number of homozygotes in the Slovenian genomic database; MAF, minor allele frequency; 2A, variants within known pharmacogenes that are more likely to have a functional significance.

| **Gene** | **Variant type** | **Transcript level variant** | **Functional effect** | **dbSNP** | **MAF**  **_gnomAD_** | **Het_Slo_**  **MAF_Slo_** | **Hom_Slo_** | **Type** | **PharmGKB** | **Drugs** | | **Phenotypes** | **ClinVar**  **(October 2017)** |
| --- | --- | --- | --- | --- | --- | --- | --- | --- | --- | --- | --- | --- | --- |
| *NAT2* | Synonymous | c.282C>T | Tyr94Tyr | 1041983 | 0.341 | 739  0.289 | 180 | Toxicity/ADR | 2A | ethambutol, isoniazid, pyrazinamide, rifampin | | Tuberculosis | 6 |
| *ADRB2* | Missense | c.46G>A | Gly16Arg | 1042713 | 0.421 | 879  0.376 | 276 | Efficacy | 2A | salbutamol, salmeterol | | Asthma | 6\|6 |
| *ABCB1* | Synonymous | c.3435T>C | Ile1145Ile | 1045642 | 0.502 | 912  0.461 | 422 | Toxicity/ADR | 2A | nevirapine | | HIV Infections | 2\|6\|6\|6\|6\|6\|6\|6\|6\|6\|6\|1 |
|  | | | | | | | | Efficacy | 2A | ondansetron | | - |  |
|  |  |  |  |  |  |  |  | Other | 2A | digoxin | | - |  |
| *CYP2C8* | Missense | c.1196A>G | Lys399Arg | 10509681 | 0.0838 | 341  0.104 | 28 | Dosage | 2A | rosiglitazone | | - | 6 |
| *CYP2C9* (*3) | Missense | c.1075A>C | Ile359Leu | 1057910 | 0.0636 | 247  0.0691 | 8 | Toxicity/ADR | 2A | antiinflammatory  agents,  non-steroids,  celecoxib,  diclofenac | | - | 6\|6\|6\|6\|6\|6\|6\|6\|6\|6\|6\|255 |
|  | | | | | | | | Dosage,Toxicity/ADR | 2A | acenocoumarol | | Haemorrhage |  |
|  |  |  |  |  |  |  |  | Dosage | 2A | celecoxib | | - |  |
| *SLCO1B1* | Intron variant | c.1865+4846T>C | - | 11045879 | . | 1  **0.000263** | 0 | Toxicity/ADR | 2A | methotrexate | | Precursor Cell Lymphoblastic Leukemia-Lymphoma | 6 |
| *CYP2D6* (*3) | Frameshift | c.775delA | Arg259fs | 35742686 | 0.0124 | 67  0.0186 | 2 | Efficacy, Toxicity/ADR | 2A | oxycodone | | Pain | 6 |
|  | | | | | | | | Other | 2A | tolterodine | | Other |  |
|  |  |  |  |  |  |  |  | Efficacy, Toxicity/ADR | 2A | mirtazapine | | - |  |
|  |  |  |  |  |  |  |  | Efficacy, Toxicity/ADR | 2A | venlafaxine | | Depressive Disorder, Obsessive-Compulsive Disorder |  |
|  |  |  |  |  |  |  |  | Other | 2A | desipramine | | Mental Disorders |  |
|  |  |  |  |  |  |  |  | Efficacy, Toxicity/ADR | 2A | atomoxetine | | Attention Deficit Disorder with Hyperactivity |  |
|  |  |  |  |  |  |  |  | Dosage | 2A | imipramine | | Depressive Disorder, Major |  |
|  |  |  |  |  |  |  |  | Metabolism/PK | 2A | metoprolol | | Hypertension |  |
|  |  |  |  |  |  |  |  | Other | 2A | flecainide | | Arrhythmias, Cardiac |  |
|  |  |  |  |  |  |  |  | Other | 2A | risperidone | | Psychotic Disorders, Schizophrenia |  |
| *CFTR* | Stop  gained | c.1624G>T | Gly542* | 113993959 | **0.000358** | 1  **0.000263** | 0 | Efficacy | 2A | ataluren | | Cystic Fibrosis | 5\|5\|5\|6 |
| *IFNL3* | Downstream gene variant | c.*814T>C | - | 11881222 | **0.00236** | **10**  **0.00262** | 0 | Efficacy | 2A | peginterferon alfa-2a,  peginterferon alfa-2b, ribavirin | | Hepatitis C, HIV | / |
| *HMGCR* | Intron variant | c.451-174A>T | - | 17244841 | . | 97  0.0286 | 6 | Efficacy | 2A | hmg coa reductase  inhibitors,  pravastatin,  simvastatin | | - | 6\|6\|6 |
| *VKORC1* | Intron variant | c.173+525C>T | - | 17708472 | . | 62  0.0215 | 10 | Dosage | 2A | warfarin | | - | 6 |
| *NAT2* | Missense | c.590G>A | Arg197Gln | 1799930 | 0.273 | 731  0.274 | 157 | Toxicity/ADR, Metabolism/PK | 2A | ethambutol, isoniazid, pyrazinamide, rifampin | | Tuberculosis | 6\|6 |
| *NQO1* | Missense | c.559C>T | Pro187Ser | 1800566 | 0.252 | 590  0.190 | 66 | Efficacy | 2A | alkylating agents, anthracyclines and related substances, fluorouracil, platinum compounds | | Breast Neoplasms, Carcinoma, Non-Small-Cell Lung, Neoplasms, Ovarian Neoplasms, Stomach Neoplasms | 255\|255\|5\|6\|1 |
| *MTHFR* | Missense | c.665C>T | Ala222Val | 1801133 | 0.314 | 857  0.371 | 277 | Toxicity/ADR | 2A | cyclophosphamide | | - | 255\|0\|255\|6\|6\|3\|6\|1\|2 |
|  | | | | | | | | Efficacy | 2A | carboplatin | | Carcinoma, Non-Small-Cell Lung |  |
|  |  |  |  |  |  |  |  | Dosage,Efficacy, Toxicity/ADR | 2A | methotrexate | | Neoplasms |  |
| *ABCB1* | Missense | c.2677T>A | Ser893Thr | 2032582 | 0.0376 | 40  0.0105 | 0 | Efficacy | 2A | simvastatin | | Hypercholesterolemia | 2\|1 |
|  | | | | | | | | Efficacy | 2A | ondansetron | | - |  |
| *CYP4F2* | Missense | c.1297G>A | Val433Met | 2108622 | 0.274 | 759  0.274 | 142 | Dosage | 2A | phenprocoumon | | - | 6\|6\|6 |
|  | | | | | | | | Other | 2A | warfarin | Over-anticoagulation | |  |
|  |  |  |  |  |  |  |  | Dosage | 2A | acenocoumarol | Atrial Fibrillation | |  |
| *CYP2B6* | Missense | c.785A>G | Lys262Arg | 2279343 | 0.126 | 344  0.133 | 82 | Metabolism/PK | 2A | efavirenz | | HIV | 6\|6 |
|  | | | | | | | | Metabolism/PK | 2A | efavirenz | | HIV, Tuberculosis |  |
| *VKORC1* | Intron variant | c.173+324T>G | - | 2884737 | 0.192 | 258  0.108 | 77 | Dosage | 2A | warfarin | | - | 6 |
| *GSTP1* | Missense | c.313A>G | Ile105Val | 1695 | 0.339 | 784  0.304 | 187 | Efficacy | 2A | fluorouracil, oxaliplatin | | Colorectal Neoplasms | 6\|6\|6\|1 |
|  | | | | | | | | Efficacy,Toxicity/ADR | 2A | cyclophosphamide,epirubicin | | Breast Neoplasms |  |
|  |  |  |  |  |  |  |  | Toxicity/ADR | 2A | platinum compounds | | Neoplasms |  |
| *CYP2B6* | Missense | c.516G>T | Gln172His | 3745274 | 0.272 | 651  0.224 | 101 | Other | 2A | nevirapine | | HIV Infections | 6\|6\|6\|6\|6 |
|  | | | | | | | | Dosage | 2A | methadone | | Heroin Dependence |  |
|  |  |  |  |  |  |  |  | Toxicity/ADR | 2A | efavirenz | | HIV |  |
| *CYP2D6* | Splice acceptor | c.506-1G>A | - | 3892097 | 0.171 | 487  0.167 | 74 | Efficacy,Toxicity/ADR | 2A | tamoxifen | | Breast Neoplasms | 6\|6\|6\|6\|6\|6\|6\|6\|6\|6\|255 |
| *UGT1A1* | Missense | c.211G>A | Gly71Arg | 4148323 | 0.0221 | 6  0.00158 | 0 | Other | 2A | SN-38 | | Neoplasms | 255\|5\|255\|255\|4\|6\|6 |
|  | | | | | | | | Other | 2A | irinotecan | | Neoplasms |  |
| *SLCO1B1* | Missense | c.521T>C | Val174Ala | 4149056 | 0.133 | 585  0.192 | 73 | Toxicity/ADR | 2A | cerivastatin | | Rhabdomyolysis | 6\|6\|6\|6\|6\|2\|255\|6 |
|  | | | | | | | | Other | 2A | rosuvastatin | | Hypercholesterolemia |  |
|  |  |  |  |  |  |  |  | Metabolism/PK | 2A | pravastatin | | - |  |
|  |  |  |  |  |  |  |  | Toxicity/ADR, Metabolism/PK | 2A | hmg coa reductase inhibitors | | Muscular Diseases, Rhabdomyolysis |  |
| *CYP2C19* | Synonymous | c.681G>A | Pro227Pro | 4244285 | 0.176 | 411  0.126 | 35 | Efficacy | 2A | citalopram | | - | 6\|6\|6\|6\|6\|6\|6\|255 |
|  | | | | | | | | Efficacy | 2A | clomipramine | | - |  |
| *COMT* | Missense | c.472G>A | Val158Met | 4680 | 0.463 | 942  0.486 | 454 | Efficacy | 2A | nicotine | | Tobacco Use Disorder | 2\|6\|2 |
| *F5* | Missense | c.1601A>G | Gln534Arg | 6025 | 0.980 | 73  0.884 | 1647 | Toxicity/ADR | 2A | hormonal contraceptives for systemic use | | Thrombosis | 6 |
| *VKORC1* | Missense | c.106G>T | Asp36Tyr | 61742245 | **0.00239** | 8  **0.00210** | 0 | Dosage | 2A | warfarin | | - | 5\|6 |
| *VDR* | Start lost | c.2T>C | Met1? | 2228570 | 0.629 | 874  0.528 | 569 | Efficacy | 2A | peginterferon alfa-2b,  ribavirin | | Hepatitis C, Chronic | 2 |
| *CYP2C9* | Missense | c.449G>A | Arg150His | 7900194 | **0.00421** | **1**  **0.000263** | 0 | Dosage, Toxicity/ADR | 2A | warfarin | | Atrial Fibrillation, Pulmonary Embolism, Stroke, Venous Thrombosis | 6 |
| *TYMS* | 3_prime UTR variant | c.*450_*455delAAGTTA | - | 151264360 | . | 115  0.0475 | 33 | Efficacy | 2A | capecitabine, fluorouracil | | Neoplasms | 6\|6 |
| *CYP3A5* | Splice acceptor&intron variant | c.-253-1G>A | - | 776746 | . | 54  0.0158 | 3 | Dosage | 2A | sirolimus | | Transplantation | 6\|6\|6\|6\|6 |

**Supplementary Table S4.** Variant-drug pairs with 2B clinical annotation according to Pharmacogenomics Knowledgebase (PharmGKB). ADR, adverse drug reaction; PK, pharmacokinetics; Het_Slo_, number of heterozygotes in the Slovenian genomic database; Hom_Slo_, number of homozygotes in the Slovenian genomic database; MAF, minor allele frequency; 2B, variant-drug pairs with moderate evidence of an association that has been replicated, but the results might not be statistically significant or the effect size may be small.

| **Gene** | **Variant type** | **Transcript level variant** | **Functional effect** | **dbSNP** | **MAF**  **_gnomAD_** | **Het_Slo_**  **MAF_Slo_** | **Hom_Slo_** | **Type** | **PharmGKB** | **Drugs** | **Phenotypes** | **ClinVar**  **(October 2017)** |
| --- | --- | --- | --- | --- | --- | --- | --- | --- | --- | --- | --- | --- |
| *FCGR3A* | Missense | c.634T>G | Phe212Val | 396991 | 0.325 | 850  0.356 | 253 | Efficacy | 2B | rituximab | Lymphoma,  B-Cell,  Lymphoma, Follicular, Lymphoma, Large-Cell, Diffuse, Lymphoma, Non-Hodgkin | 6\|6\|6\|3 |
|  | | | | | | | | Efficacy | 2B | trastuzumab | Breast Neoplasms |  |
|  |  |  |  |  |  |  |  | Efficacy | 2B | cetuximab | Colorectal Neoplasms |  |
| *TP53* | Missense | c.215C>G | Pro72Arg | 1042522 | 0.669 | 703  0.718 | 1016 | EfficacyToxicity/ADR | 2B | antineoplastic agents, cisplatin, cyclophosphamid, fluorouracil, paclitaxel | Breast Neoplasms, Neoplasms, Neutropenia, Ovarian Neoplasms, Stomach Neoplasms | 2\|2\|2\|2\|0\|6\|6\|6\|6\|6\|2 |
| *ABCB1* | Synonymous | c.3435T>C | Ile1145Ile | 1045642 | 0.502 | 912  0.461 | 422 | Dosage | 2B | fentanyl, methadone, morphine, opioids, oxycodone, tramadol | Pain | 2\|6\|6\|6\|6\|6\|6\|6\|6\|6\|6\|1 |
| *EPHX1* | Missense | c.337T>C | Tyr113His | 1051740 | 0.321 | 792  0.303 | 180 | Dosage | 2B | carbamazepine | Epilepsy | 255\|255\|255\|255\|6 |
| *CBR3* | Missense | c.730G>A | Val244Met | 1056892 | 0.367 | 885  0.369 | 261 | Toxicity/ADR | 2B | anthracyclines and related substances | Heart Failure, Neoplasms | 6 |
| *DRD2* | Intron  variant | c.811-83G>T | - | 1076560 | . | 300  0.0945 | 30 | Toxicity/ADR | 2B | cocaine | Cocaine-Related Disorders | 6 |
| *ITPA* | Missense | c.94C>A | Pro32Thr | 1127354 | 0.0750 | 270  0.0762 | 10 | Dosage,Toxicity/ADR | 2B | interferon alfa-2b, recombinant, ribavirin | Hepatitis C, Chronic | 255\|6\|6 |
|  | | | | | | | | Toxicity/ADR | 2B | peginterferon alfa-2b,  ribavirin | Hepatitis C, Chronic |  |
| *ERCC1* | Synonymous | c.354T>C | Asn118Asn | 11615 | 0.499 | 841  0.376 | 296 | EfficacyToxicity/ADR | 2B | carboplatin, cisplatin, oxaliplatin, platinum, Platinum compounds | Carcinoma, Non-Small-Cell Lung, Colorectal Neoplasms, Esophageal Neoplasms, Mesothelioma, Neoplasms, Ovarian Neoplasms, Pancreatic Neoplasms | 6\|6\|6\|6\|6\|2 |
| *HTR2C* | Intron  variant | c.551-3008C>G | - | 1414334 | . | 0  **0.00158** | 3 | Toxicity/ADR | 2B | antipsychotics, clozapine, risperidone | Metabolic Syndrome X,  Schizophrenia | 6\|6\|6 |
| *ABCC4* | Synonymous | c.3348G>A | Lys1116Lys | 1751034 | 0.810 | 577  0.770 | 1178 | Metabolism/PK | 2B | tenofovir | HIV,  HIV Infections | 6 |
| *OPRM1* | Missense | c.397A>G | Asn133Asp | 1799971 | 0.188 | 434  0.131 | 32 | Efficacy | 2B | naloxone | - | 0\|6\|6\|6\|6\|6\|6\|6\|6\|6\|6 |
|  | | | | | | | | Toxicity/ADR | 2B | ethanol | - |  |
|  |  |  |  |  |  |  |  | Metabolism/PK | 2B | alfentanil,buprenorphine, fentanyl, heroin, morphine, opioids, sufentanil, tramadol | Heroin Dependence,  Opioid-Related Disorders,  Pain,  Pain, Postoperative |  |
| *ANKK1* | Missense | c.2137G>A | Glu713Lys | 1800497 | 0.264 | 546  0.173 | 56 | Toxicity/ADR | 2B | antipsychotics, clozapine, olanzapine, risperidone | Hyperprolactinemia,  tardive dyskinesia,  Weight gain | 5\|6\|6\|6\|6\|6\|6 |
|  | | | | | | | | Toxicity/ADR | 2B | ethanol | Alcoholism |  |
| *UMPS* | Missense | c.638G>C | Gly213Ala | 1801019 | 0.195 | 536  0.173 | 61 | Toxicity/ADR | 2B | capecitabine, fluorouracil, leucovorin, tegafur | Drug Toxicity | 1\|6\|6\|6\|6\|2\|2 |
| *MTRR* | Missense | c.147A>G | Ile49Met | 1801394 | 0.468 | 879  0.547 | 602 | Toxicity/ADR | 2B | methotrexate | Precursor Cell Lymphoblastic Leukemia-Lymphoma | 255\|255\|2\|0\|6\|2 |
| *CRHR1* | Intron  variant | c.1194 +111C>T | - | 1876828 | . | 497  0.159 | 55 | Efficacy | 2B | budesonide, corticosteroids, fluticasone propionate, fluticasone/salmeterol, triamcinolone | Asthma | 6\|6\|6\|6\|6 |
| *UGT2B15* | Missense | c.253T>G | Tyr85Asp | 1902023 | 0.512 | 861  0.446 | 418 | Other | 2B | lorazepam,  oxazepam | - | 6\|6 |
| *GRIK4* | Intron  variant | c.83-10039T>C | - | 1954787 | . | 2  **0.00525** | 9 | Efficacy | 2B | antidepressants | Depression, Depressive Disorder,  Depressive Disorder, Major | 6\|6 |
| *UGT1A4* | Missense | c.142T>G | Leu48Val | 2011425 | 0.114 | 316  0.0877 | 9 | Other | 2B | lamotrigine | Epilepsy | 6 |
| *ABCG2* | Missense | c.421C>A | Gln141Lys | 2231142 | 0.124 | 371  0.107 | 18 | Efficacy, Dosage | 2B | allopurinol | Gout | 255\|255\|6\|6\|1 |
|  | | | | | | | | Efficacy | 2B | rosuvastatin | Hypercholesterolemia,  Myocardial Infarction |  |
| *EPHX1* | Missense | c.416A>G | His139Arg | 2234922 | 0.187 | 604  0.199 | 77 | Dosage | 2B | carbamazepine | Epilepsy | 2\|6 |
| *ADORA2A* | Upstream  gene variant | c.-631C>T | - | 2298383 | . | 1  **0.00131** | 2 | Toxicity/ADR | 2B | caffeine | - | - |
| *XRCC1* | Missense | c.1196A>G | Gln399Arg | 25487 | 0.681 | 819  0.625 | 780 | EfficacyToxicity/ADR | 2B | carboplatin,  cisplatin, oxaliplatin, platinum, Platinum compounds | Carcinoma, Non-Small-Cell Lung, Colorectal Neoplasms, Neoplasms, Ovarian Neoplasms, Pancreatic Neoplasms, Uterine Cervical Neoplasms | 6\|6\|6\|6\|6 |
| *SCN1A* | Intron  variant | c.603-91G>A | - | 3812718 | . | 834  0.511 | 556 | Dosage | 2B | phenytoin | Epilepsy | 255\|6\|6\|6\|6 |
|  | | | | | | | | Dosage | 2B | carbamazepine | Epilepsy |  |
|  |  |  |  |  |  |  |  | Efficacy | 2B | antiepileptics, carbamazepine | Epilepsy |  |
| *EGF* | 5_prime UTR variant | c.-382A>G | - | 4444903 | . | 10  **0.00630** | 7 | Efficacy | 2B | cetuximab | Colorectal Neoplasms, Rectal Neoplasms | 6\|2 |
| *ATIC* | Intron  variant | c.1503+675T>C | - | 4673993 | 0.341 | 127  0.0538 | 39 | Efficacy | 2B | methotrexate | Arthritis, Rheumatoid | 6 |
| *COQ2* | Intron  variant | c.779-1022C>G | - | 4693075 | . | 1  **0.000263** | 0 | Toxicity/ADR | 2B | atorvastatin, hmg coa reductase inhibitors, rosuvastatin | Muscular Diseases | 6\|6\|6 |
| *FKBP5* | Intron  variant | c.-20+18122T>C | - | 4713916 | . | 2  **0.00158** | 2 | Efficacy | 2B | antidepressants, citalopram, fluoxetine, mirtazapine, paroxetine, Selective serotonin reuptake inhibitors, venlafaxine | Depressive Disorder, Major, Mood Disorders | 6\|6\|6\|6\|6\|6\|6 |
| *CYP2B6* | Intron  variant | c.485-18C>T | - | 4803419 | 0.339 | 783  0.308 | 195 | Metabolism/PK | 2B | efavirenz | HIV | 6 |
| *SOD2* | Missense | c.47T>C | Val16Ala | 4880 | 0.485 | 880  0.480 | 473 | Efficacy | 2B | cyclophosphamide | Breast Neoplasms | 2\|255\|6 |
| *ADD1* | Missense | c.1378G>T | Gly460Trp | 4961 | 0.203 | 555  0.172 | 50 | Efficacy | 2B | furosemide, spironolactone | Liver Cirrhosis | 255\|6 |
| *GNB3* | Synonymous | c.825C>T | Ser275Ser | 5443 | 0.354 | 809  0.317 | 200 | Efficacy | 2B | sildenafil | Erectile Dysfunction | 6 |
| *UGT2B10* | Missense | c.199G>T | Asp67Tyr | 61750900 | 0.0652 | 250  0.0730 | 14 | Metabolism/PK | 2B | nicotine | - | 6 |
| *CES1* | Missense | c.431G>A | Gly144Glu | 71647871 | 0.0108 | 53  0.0139 | 0 | Efficacy | 2B | clopidogrel | - | 6 |
| *ITPA* | Intron variant | c.124+21A>C | - | 7270101 | 0.0873 | 397  0.122 | 33 | Toxicity/ADR | 2B | peginterferon alfa-2b, ribavirin | Hepatitis C, Chronic | 255\|6\|6\|6 |
| *SLC28A3* | Synonymous | c.1381C>T | Leu461Leu | 7853758 | 0.152 | 452  0.141 | 43 | Toxicity/ADR | 2B | anthracyclines and related substances | Neoplasms | 6 |
| *HTR2A* | Intron variant | c.614-2211T>C | - | 7997012 | . | 2  **0.00105** | 1 | Efficacy | 2B | antidepressants, citalopram, selective serotonin reuptake inhibitors | Depression, Depressive Disorder | 6\|6\|6 |
| *SLC28A3* | Intron variant | c.862-360C>T | - | 885004 | . | 128  0.0420 | 16 | Toxicity/ADR | 2B | anthracyclines and related substances | Neoplasms | 6 |
| *SLC47A2* | 5_prime UTR variant | c.-130C>T | - | 12943590 | **.** | 648  0.237 | 127 | Efficacy | 2B | metformin | Diabetes Mellitus | 6 |
| *GSTP1* | Missense | c.313A>G | Ile105Val | 1695 | 0.339 | 784  0.304 | 187 | Toxicity/ADR | 2B | cisplatin | Medulloblastoma, Testicular Neoplasms | 6\|6\|6\|1 |
| *COMT* | Missense | c.472G>A | Val158Met | 4680 | 0.463 | 942    0.486 | 454 | Dosage, Efficacy | 2B | methadone, morphine, opioids, oxycodone, sufentanil, tramadol | Low Back Pain, Pain | 2\|6\|2 |
